# Prospective Association of Circulating Adipokines with Cardiometabolic Risk Profile Among Women: The Rape Impact Cohort Evaluation Study

**DOI:** 10.1089/whr.2022.0069

**Published:** 2022-10-07

**Authors:** Eileen Vuong, Nasheeta Peer, Esnat Chirwa, Shibe Mhlongo, Carl Lombard, Sian Hemmings, Andre Pascal Kengne, Naeemah Abrahams, Soraya Seedat

**Affiliations:** ^1^ South African Research Chairs Initiative (SARChI), PTSD Program, Department of Psychiatry, Stellenbosch University, Stellenbosch, South Africa.; ^2^ Department of Psychiatry, Stellenbosch University, Stellenbosch, South Africa.; ^3^ Non-Communicable Diseases Research Unit, South African Medical Research Council, Tygerberg, South Africa.; ^4^ Department of Medicine, University of Cape Town, Cape Town, South Africa.; ^5^ Gender and Health Research Unit, South African Medical Research Council, Tygerberg, South Africa.; ^6^ School of Public Health, Faculty of Health Sciences, University of Witwatersrand, Johannesburg, South Africa.; ^7^ Biostatistics Unit, South African Medical Research Council, Tygerberg, South Africa.; ^8^ SAMRC/SU Genomics of Brain Disorders Unit, Stellenbosch University, South Africa.; ^9^ Faculty of Health Sciences, School of Public Health and Family Medicine, University of Cape Town, Cape Town, South Africa.

**Keywords:** adipokines, cardiometabolic, rape, South Africa

## Abstract

**Background:**

Sexual violence is associated with poor cardiometabolic outcomes, yet the etiopathogenic pathways remain unclear. Adipokines may contribute to pathways in the development of cardiometabolic disease (CMD), including in vulnerable populations. Further investigation of adipokines among sexually traumatized individuals may inform cardiometabolic screening. This study aimed to investigate the association between circulating adipokines, metabolic syndrome (MetS), and longitudinal change in MetS components (namely abdominal obesity, blood pressure, lipid profile, and glycemic status) over a 1-year period in a cohort of rape exposed (RE) and rape unexposed (RUE) females.

**Materials and Methods:**

Seven hundred seventy-eight RE and 617 RUE black South African women aged 18–40 years were recruited for the Rape Impact Cohort Evaluation study. Nonfasting blood samples were analyzed for cardiometabolic variables and adipokine levels using enzyme-linked immunosorbent assay. Serum adiponectin was measured in both RE and RUE and resistin, leptin, and leptin/adiponectin (L/A) ratio in RE only. Associations between baseline serum adipokines, MetS, and its components were assessed at baseline and follow-up using adjusted linear and logistic regressions.

**Results:**

In the RE group, adiponectin, leptin, and L/A ratio were significantly associated with MetS prevalence cross-sectionally (all *p* ≤ 0.001). No adipokine marker was related to incident MetS at 12-month follow-up. In the RE group, significant longitudinal associations with high-density lipoprotein cholesterol were shown for adiponectin (β = 0.146 [0.064], *p* = 0.022) and leptin (β = 0.001 [0.002], *p* = 0.012).

**Conclusions:**

Findings suggest that adipokines may have a potential role as biomarkers to identify RE individuals at high risk for CMD.

## Introduction

Sexual violence, including rape, is a major global public health concern, disproportionately affecting women.^1,2^ In South Africa, the lifetime prevalence of rape against women is among the highest globally, with estimates ranging from 12% to 25%.^1,3–5^ Rape is associated with a broad range of potential adverse physical and mental health consequences. Some of the consequences of rape are immediate and direct, for example, unwanted pregnancy, sexually transmitted infections, and physical injuries.^6^ However, sexual violence can also increase the risk of future ill health, including the risk of cardiometabolic diseases (CMDs).^7–9^ While there is a great deal of consensus that rape is detrimental to physical health, the pathophysiologic link between sexual violence and CMDs remains unclear.

It has been hypothesized that rape triggers an acute and sustained elevated stress response, compromising the neuroendocrine and immune systems, leading to an increased cardiometabolic risk.^10,11^ In general, biological studies in adult females who have been sexually assaulted are scarce.^12,13^ An improved understanding of the pathophysiological processes that increase cardiometabolic risk following sexual violence may inform early identification, prevention, and treatment efforts.^13^

Among the novel biomarkers under investigation for identifying individuals at high risk for CMDs are adipokines. Adipokines are fat-secreted cytokines with diverse signaling effects that modulate insulin resistance, hepatic lipoprotein production, and vascular inflammation.^14^ Adiponectin, the most abundant adipokine, exerts anti-inflammatory, insulin-sensitizing, and anti-atherogenic properties.^15^ Low adiponectin levels are associated with type 2 diabetes, obesity, metabolic syndrome (MetS), and coronary artery disease.^16–18^ In contrast, high serum concentrations of pro-inflammatory adipokines, such as leptin and resistin, are found with obesity and other CMDs.^19,20^ Given the opposing effects of adiponectin and leptin on glucose and fat metabolism, the leptin/adiponectin (L/A) ratio has been proposed as a better marker of adipose tissue dysfunction and insulin resistance than adiponectin or leptin individually.^21^

Adipokine dysregulation following rape may be a marker for future risk of CMDs. The ability to identify rape-exposed (RE) women at greatest risk of CMDs early could allow for the implementation of interventive and preventive strategies when the disease trajectory may still be modifiable. This is particularly pertinent in South Africa where CMDs among women are prevalent^22^ and contribute to significant health care costs, morbidity, and mortality.^23^

Posttraumatic stress disorder (PTSD) and depression are other common long-term health sequelae of sexual assault.^24,25^ To our knowledge, no longitudinal studies exploring the relationships between adipokines and CMDs in general populations have considered nontraditional cardiometabolic risk factors such as PTSD, depression, and other traumatic experiences (*e.g.*, childhood abuse).^26–28^ Adipokine levels have been shown to be altered among individuals with PTSD symptoms, depression, and those who have experienced childhood maltreatment.^29–32^ Moreover, PTSD and CMDs may each activate neurobiological pathways common in both, such as the autonomic nervous system, hypothalamic pituitary adrenal (HPA) axis, and immune-mediated pathways.^33,34^ Longitudinal studies that account for nontraditional cardiometabolic risk factors may assist in unraveling the biological pathways linking sexual violence and CMDs.

In this study we address limitations of previous research by investigating the prospective association of four adipokine markers (adiponectin, leptin, resistin, and L/A ratio) and cardiometabolic profiles in a cohort of RE and rape-unexposed (RUE) female participants. We accounted in analyses for a wide range of mental health factors, which could be considered confounders and/or mediators of the association between sexual violence and CMDs. Specifically, we examined (1) cross-sectional associations between baseline adipokines and body mass index (BMI), baseline MetS prevalence and individual MetS components (namely waist circumference [WC], glycated hemoglobin [HbA1c], triglycerides, high-density lipoprotein cholesterol [HDL-C], and blood pressure [BP]); (2) longitudinal associations between baseline adipokine levels and changes in levels of MetS components at 12-month follow-up; and (3) longitudinal associations between baseline adipokine levels and incident MetS at 12 months.

We hypothesized the following: (1) high serum adiponectin (s-ADP) levels would be associated with a favorable cardiometabolic profile (namely lower BMI, lower risk of MetS, and adverse MetS components [increased WC, raised BP, dysglycemia, and dyslipidemia]) and in contrast, (2) high serum leptin levels, resistin levels, and L/A ratio would be associated with an unfavorable cardiometabolic health profile (higher BMI, higher risk of MetS, and adverse MetS components [increased WC, raised BP, dysglycemia, and dyslipidemia]), and (3) baseline s-ADP levels would be lower in RE compared to RUE participants.

## Materials and Methods

### Setting and participants

RE (*N* = 852) female participants were recruited for the parent study, “Rape Impact Cohort Evaluation (RICE),” a large prospective cohort study evaluating the impact of rape on women's health and their use of health services in South Africa.^35^ A detailed description of the methods used in the parent study has been published.^35^ In brief, female rape survivors were recruited from four rape centers located in the South African province of KwaZulu-Natal. Recruitment was restricted to female participants between 16 and 40 years of age who reported a rape within the previous 20 days. The South African Criminal Law (Sexual Offences and Related Matters) Amendment Act 32 of 2007 defines rape as any unlawful and intentional form of oral, anal, or vaginal penetration without consent, irrespective of gender. Women recruited into this study all reported vaginal penetration. Informed consent was obtained by research assistants trained in conducting research in vulnerable populations.

A control group of RUE participants (*N* = 947) was drawn from women attending Primary Health Care clinics, mainly Family Planning and Child Wellness services. Women who reported previous (lifetime) exposure to sexual violence, other than the recent rape (within 20 days), were identified with three screening questions at the baseline interview. Participants who indicated one/more of the following: intimate partner rape and/or nonpartner rape and/or first sexual intercourse that was forced/rape; were excluded from the unexposed cohort. Recruitment took place between October 2014 and April 2019 (42 months). Participants in both the case and control groups were excluded if they were too emotionally distressed at the time of enrolment to provide informed consent, intellectually disabled, or more than 14 weeks pregnant.

**FIG. 1.**
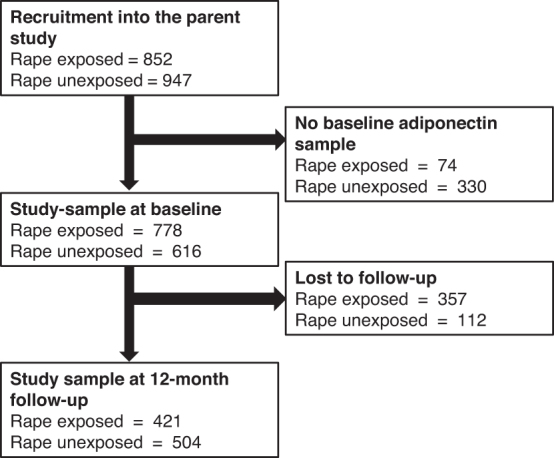
Participant selection.

After enrolment, participant completed baseline assessments within 20 days postrape. Follow-up assessments were completed every 3 months in the 1st year and every 6 months thereafter. For the current substudy, we included all RE and RUE participants with (1) complete clinical datasets for the two time points of interest (baseline: RE = 778, RUE = 616, and 12 months: RE = 421, RUE = 504) and (2) a blood sample for adipokine testing at baseline (Fig. 1).

### Data collection

Information on sociodemographic characteristics, health behavioral factors, mental health, and medical history was collected from study participants at baseline and 12-month follow-up by trained fieldworkers using standardized protocols. In this study, all self-reported mental health assessments were administered by trained research assistants under the supervision of a registered trauma counselor or nurse.

### Behavioral risk factors

#### Alcohol Use Disorders Identification Test-C

The Alcohol Use Disorders Identification Test-C (AUDIT-C), a subscale of the original 10-item AUDIT screening tool developed by the World Health Organization was administered to assess for problematic drinking.^36^ A cutoff of three indicates hazardous drinking.

#### Smoking status

Smoking was assessed through two questions on daily and occasional smoking. Daily smoking was defined as smoking ≥1 cigarette per day. Occasional smoking was defined as smoking cigarettes, but not daily.

### Mental health factors

#### Posttraumatic stress disorder

The *Mini International Neuropsychiatric Interview*, version 7.0.0, a structured psychiatric interview that screens for 16 Diagnostic and Statistical Manual of Mental Disorders, 5th Edition (DSM-5) psychiatric disorders, was administered at the baseline visit to assess for a diagnosis of PTSD based on past trauma (*i.e.*, trauma before the index rape).^37^

The *Davidson Trauma Scale*, a 17-item self-report questionnaire of posttraumatic stress symptoms, was administered at all timepoints and used to identify PTSD at the 3- and 6-month follow-up visits, using a cutoff score ≥40.^38^ RE participants completed the DTS in relation to the recent rape event.

### Past trauma experiences

A modified version of the *Life Events Checklist (LEC)* was used to measure lifetime exposure to different trauma types at the baseline visit.^5,39^ The modified version of the LEC measures direct exposure to 10 trauma types using a dichotomous “yes/no” response. The trauma types measured were (1) imprisonment, (2) civil unrest/war, (3) serious injury, (4) being close to death, (5) murder of a family member or friend, (6) unnatural death of a family member or friend, (7) murder of a stranger/s, (8) robbed at gunpoint or knifepoint, (9) kidnapping, and (10) sexual assault. An item from the modified version (“torture”) was not completed accurately by participants. Torture is not well translated in isiZulu, and the meaning of “torture” was therefore misunderstood and misinterpreted. This item was therefore excluded from calculation of the total score on the LEC-modified version. The number of “yes” responses from the remaining trauma types were added together to yield a total LEC score ranging from 0 to 10, indicating the cumulative lifetime trauma load of participants.

Childhood trauma (before the age of 18 years) was measured using a modified version of the *Childhood Trauma Questionnaire-Short Form (CTQ-SF)*.^40,41^ The modified version of the CTQ-SF consists of 14 items and measures five types of childhood trauma, namely (1) witness of abuse of mother, (2) sexual abuse, (3) physical abuse, (4) emotional abuse, and (5) parental neglect. Responses were measured on a 4-point Likert scale ranging from 1 (“never”) to 4 (“very often”), total score range between 14 and 56.

#### Depression

The presence of depression at all time points was established using the *Centre for Epidemiologic Studies Depression Scale (CES-D)*. The CES-D is a 20-item, self-report measure with responses measured on a 4-point Likert scale ranging from 0 (“rarely or none of the time”) to 3 (“most or all of the time”), total score range between 0 and 60. A cutoff score of ≥16 has been recommended as indicative of caseness for depression with good sensitivity and specificity and high internal consistency.^42^ Depression was assessed for the week preceding the baseline, 3-month, and 6-month visit.

### Clinical and anthropometric measurements

Clinical and anthropometric measurements were recorded at the baseline and 12-month follow-up. BP was measured using a digital BP monitor (Omron, M6 Comfort, Netherland) with participants seated for at least 5 minutes before measurements. Three measurements were taken 3 minutes apart, and the average of the second and third readings was used in the analysis. We included three anthropometric indices, namely BMI, WC, and waist-to-hip ratio (WHR) as proxy indicators of generalized and central obesity, respectively.

Height, weight, WC, and hip circumference (HC) were obtained using standard techniques. Height was measured using a stadiometer in a standing upright position without shoes. Weight was measured on a calibrated scale with participants in light clothing, without shoes, and recorded to nearest 0.5 kg. BMI was calculated as weight (in kilograms) divided by height (in square meters).^43^ WC was measured in centimeters using a nonelastic tape measure at the end of a normal expiration at the midpoint between the lower border of the lowest rib and the upper border of the hip bone, with the measuring tape parallel to the floor. WC was measured to the nearest 0.1 cm. Participants' BMI was categorized as normal (≤24.9 kg/m^2^), overweight (25–29.9 kg/m^2^), or obese (≥30 kg/m^2^). HC was measured around the widest portion of the buttocks, with the measuring tape parallel to the floor. WHR was calculated as WC divided by HC.

### Biochemical analyses

At the baseline and 12-month visits, nonfasting venous blood samples were collected in the morning by a trained research nurse. Samples were kept on ice and sent to the testing laboratory within 2–4 hours. Whole blood fractions were separated by centrifuging the sample for 5 minutes at 3000 rpm at 4°C, aliquoted into individual tubes, and stored at −80°C without freeze–thaw cycles until the day of analysis. All blood samples were analyzed by a single laboratory following a standardized protocol.

Adiponectin assays were performed on the blood samples of all RE and RUE participants, while leptin and resistin assays, due to their high costs, were performed on the blood samples of RE participants only. Circulating s-ADP, leptin, and resistin levels were measured using a commercially available quantitative sandwich Enzyme-linked Immunosorbent Assay (ELISA) Kit (Quantikine, R&D Systems Inc., Minneapolis, MN). All procedures were performed in strict accordance with the manufacturer's instructions. The L/A ratio was calculated by dividing the absolute value of serum leptin (ng/mL) by the absolute value of s-ADP (μg/mL). The average inter- and intra-assay coefficients of variations were 5.5% and 6.0% for adiponectin; 7.4% and 8.1% for leptin; and 4.6% and 6.9% for resistin, respectively.

All samples were analyzed for total cholesterol, triglycerides, low-density lipoprotein cholesterol, HDL-C, HbA1c, and HIV infection (ELISA test). Lipid profiles and HbA1c were measured using enzymatic methods.

### Outcome definitions

#### Metabolic syndrome

The prevalence and incidence of MetS were ascertained at baseline and 12-month follow-up, respectively. MetS was defined according to modified Joint Interim Statement criteria^44^ as the presence of at least three of five cardiometabolic abnormalities: (1) elevated WC (≥80 cm); (2) elevated nonfasting triglyceride levels (≥1.7 mmol/L); (3) reduced HDL-C (≤1.3 mmol/L); (4) elevated BP (systolic blood pressure [SBP] ≥130 mmHg or diastolic blood pressure [DBP] ≥85 mmHg) or previously diagnosed with hypertension as assessed at baseline; and (5) impaired glucose tolerance (HbA1c ≥5.7% or ≥39 mmol/L) or self-reported diabetes.^45^

#### Longitudinal change of MetS components at follow-up

We evaluated changes in the levels of the MetS components between the baseline and the 12-month follow-up period. The MetS components evaluated were WC, SBP and DBP, triglycerides, HDL-C, and HbA1c.

### Ethical statement

All participants provided written informed consent. Ethical approval for the RICE study was obtained from the South African Medical Research Council Ethics Committee (SAMRC; EC019/2013). Approval to conduct the substudy was obtained from the Health Research Ethics Committee at Stellenbosch University in Cape Town, South Africa (S17/10/265).

### Statistical analyses

All data were analyzed using Stata16.0 software (Stata/SE 16.0; StataCorp LLC, College Station, TX). We performed a primary analysis, examining the associations between s-ADP and cardiometabolic outcomes for the combined study population (baseline: RE = 778, RUE = 616; 12 months: RE = 421, RUE = 504). This was followed by a secondary analysis of the remaining adipokines, namely leptin, resistin, and L/A ratio, in the RE group only.

Descriptive analyses of the baseline characteristics of the study population are presented as frequencies (percentages) for categorical variables and mean ± standard deviation (SD) or median (interquartile range) for continuous variables depending on distribution (normal vs. non-normal). To assess associations between baseline characteristics and retention status, as defined by two categories, namely dropout (baseline visit only) or completed all visits (baseline and 12 months), analysis of variance was used for continuous variables and Pearson chi-square test for categorical variables (Supplementary Table S1). The relationship between baseline serum adipokine concentrations and individual cardiometabolic components at baseline, namely WC, HDL-C, triglycerides, SBP, DBP, and HbA1c, was analyzed using linear regression models. Multiple linear regression models were performed for each component adjusted for participants' age and other appropriate covariates (HIV, alcohol use, smoking status, depression, PTSD, lifetime, and childhood trauma).

Simple and multiple linear regressions were used to examine the relationship between baseline adipokine levels and the relative change in individual cardiometabolic component level at follow-up. We calculated the relative change in individual cardiometabolic component as: 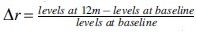. Multiple regression models were adjusted for baseline cardiometabolic factors. We also assessed if there was any significant interaction between the RE group and adipokine levels.

Finally, we used multivariable logistic regressions to relate the entire panel of adipokines to incident MetS at 12 months, in participants without MetS at the baseline examination. We adjusted for baseline cardiometabolic factors, age, smoking status, alcohol consumption, depression status at 12-month follow-up, PTSD status at 12-month follow-up, lifetime trauma, and HIV status. The interaction effect of rape exposure was also investigated.

We used variance inflation factor (>10) to assess for the presence of high multicollinearity among the metabolic variables adjusted for in the models. Furthermore, we ran multivariate linear regression models to simultaneously assess the relationship between baseline adipokines and baseline MetS and between baseline adipokines and relative change in MetS at 12 months. For all analyses, a *p*-value of <0.05 was considered statistically significant and corrected for multiple comparisons using Bonferroni correction.

### Power calculation

Sample size calculation and sampling for the parent study have been described previously.^35^ An illustrative power calculation to yield at least 80% power was done for this substudy based on the available sample size and the estimated effect size based on the known prevalence of MetS of 20%, in the young black South African female population^46,47^ (Supplementary Table S2 in Supplementary Data).

## Results

### Cohort description

The main sociodemographic, behavioral, and clinical characteristics at baseline are presented in Table 1. The mean age of the study cohort was 25.2 years (SD 5.4 years), and 43.8% of participants were HIV positive. More than a half (64.4%) presented with clinically significant depressive symptoms, and more than a quarter (27.2%) were found to engage in harmful alcohol use. Eighteen percent of RE participants had PTSD at baseline.

**Table 1.** Sociodemographic, Clinical, and Biochemical Characteristics of the Study Population at Baseline Across Groups

Variables	All (*N* = 1395)		RE (*n* = 778)		RUE (*n* = 617)	
	*n* (%)	Mean (SD)	*n* (%)	Mean (SD)	*n* (%)	Mean (SD)
Sociodemographic						
Age (years)		25.2 (5.4)		24.9 (5.3)		25.7 (5.4)
Completed secondary education	821 (58.85)		446 (57.33)		375 (60.78)	
Employed	294 (21.08)		203 (26.09)		91 (14.75)	
Mental health						
Depression^a^	898 (64.37)		705 (90.62)		193 (31.28)	
PTSD^b^	161 (11.54)		138 (17.74)		23 (3.73)	
Childhood trauma (CTQ)		16.2 (3.3)		16.6 (3.7)		15.8 (2.6)
Lifetime trauma (LEC)		1.7 (1.7)		2 (1.8)		1.2 (1.5)
Behavioral						
Harmful alcohol use^c^	380 (27.24)		239 (30.72)		141 (22.85)	
Smoker^d^	175 (12.54)		116 (14.91)		59 (9.56)	
Clinical						
Anthropometry						
Weight (kg)		67.8 (17.2)		65.3 (15.5)		71 (18.8)
HC (cm)		105.4 (12.6)		103.7 (11.6)		107.5 (13.5)
WC (cm)		84.4 (14.1)		82.3 (12.5)		87.1 (15.4)
WHR		0.8 (0.1)		0.8 (0.1)		0.8 (0.1)
BMI (kg/m^2^)		27 (6.5)		26 (5.9)		28.2 (7.1)
BP						
SBP (mmHg)		105 (8.7)		104.2 (8.5)		106 (8.8)
DBP (mmHg)		70.4 (7.6)		69.5 (7.4)		71.4 (7.8)
Abnormal BP, %^e^	199 (14.3)		116 (14.9)		83 (13.5)	
Heart rate (beats/min)		74.5 (8.6)		74.2 (8.6)		74.9 (8.6)
Biochemical						
Glucose						
HbA1c (%)		5.3 (0.5)		5.4 (0.5)		5.3 (0.6)
Dysglycemia^f^	308 (22.08)		186 (23.91)		122 (19.77)	
Lipids						
Total cholesterol (mmol/L)		3.8 (0.8)		3.8 (0.8)		3.9 (0.8)
Triglycerides (mmol/L)		0.8 (0.4)		0.8 (0.4)		0.8 (0.4)
HDL-C (mmol/L)		1.2 (0.3)		1.2 (0.3)		1.2 (0.3)
LDL-C (mmol/L)		2.2 (0.7)		2.2 (0.7)		2.3 (0.7)
Adipokines						
Adiponectin (μg/mL)		12.8 (5.2)		12.8 (5.9)		12.9 (4.3)
Leptin (ng/mL)				13.1 (11.1)		
Resistin (ng/mL)				14.1 (9.1)		
Leptin/adiponectin ratio				1.5 (1.9)		
HIV status						
HIV positive	611 (43.8)		369 (47.4)		242 (39.2)	

Values for continuous variables are presented as mean ± SD or as median and IQR.

^a^ Depression based on CES-D ≥16.

^b^ PTSD according to Mini Neuropsychiatric interview.

^c^ Harmful alcohol use according to AUDIT-C ≥3.

^d^ Smoker includes daily and occasional smokers.

^e^ Abnormal BP: SBP ≥130 mmHg or DBP ≥85 mmHg or self-reported hypertension.

^f^ Dysglycemia: HbA1c ≥5.7 mmol/L or self-reported diabetes.

AUDIT-C, Alcohol Use Disorders Identification Test-C; BMI, body mass index; BP, blood pressure; CES-D, Centre for Epidemiologic Studies Depression Scale; CTQ, childhood trauma questionnaire; DBP, diastolic blood pressure; HC, hip circumference; HDL-C, high-density lipoprotein cholesterol; IQR, interquartile range; LDL-C, low-density lipoprotein cholesterol; LEC, life events checklist; PTSD, posttraumatic stress disorder; RE, rape-exposed; RUE, rape-unexposed; SBP, systolic blood pressure; SD, standard deviation; WC, waist circumference; WHR, waist-to-hip ratio.

### Cross-sectional analyses

#### BMI and MetS distribution at baseline

Table 2 shows the distribution of adipokine levels across the BMI categories at baseline. In the RE group, s-ADP levels were the lowest among obese participants and leptin, resistin, and L/A ratio were the highest, compared to overweight and normal weight participants. This linear trend was not observed for the RUE group.

**Table 2.** Associations Between Body Mass Index Status and Adipokine Markers by Groups at Baseline

	All participants				RE (*N* = 778)				RUE (*N* = 617)			
	BMI <25 kg/m^2^ (*n* = 390)	BMI 25–29.9 (*n* = 211)	BMI ≥30 (*n* = 324)	*p*	BMI <25 kg/m^2^ (*n* = 390)	BMI 25–29.9 (*n* = 211)	BMI ≥30 (*n* = 324)	*p*	BMI <25 kg/m^2^ (n = 390)	BMI 25–29.9 (n = 211)	BMI ≥30 (n = 324)	*p*
Adiponectin (μg/mL)	13.6	12.4	12.0	**<0.001**	14.0	12.3	10.4	**<0.001**	12.8	12.6	13.2	0.426
Leptin (ng/mL)^a^					6.9	12.3	19.0	**<0.001**				
Resistin (ng/mL)^a^					11.7	11.9	13.5	**0.014**				
L/A ratio^a^					0.5	1.2	2.0	**<0.001**				

The BMI was categorized into normal weight (<25 kg/m^2^), overweight (25–29.9 kg/m^2^), obese (≥30 kg/m^2^). Leptin and resistin levels were only collected among the rape exposed participants. Values in bold indicate *p*-value ≤0.05.

^a^ Median values (Kruskal–Wallis test used).

L/A, leptin/adiponectin.

At baseline, 14.5% (*n* = 113) RE and 18% (*n* = 112) RUE participants met study criteria for MetS, Table 3. A greater proportion of RE compared to RUE participants had an elevated WC (*p* < 0.001).

**Table 3.** Prevalence of Cardiometabolic Abnormalities at Baseline

	All participants (*N* = 1394)	RE (*N* = 778)	RUE (*N* = 617)	*p*
	*n* (%)	*n* (%)	*n* (%)	
WC ≥80 cm				
No	638 (45.73)	407 (52.31)	231 (37.44)	**<0.001**
Yes	757 (54.27)	371 (47.69)	386 (62.56)	
Triglycerides ≥1.7 mmol/L				
No	1198 (96.07)	640 (96.39)	558 (95.71)	0.541
Yes	49 (3.93)	24 (3.61)	25 (4.29)	
HDL ≤1.3 mmol/L				
No	399 (32)	212 (31.93)	187 (32.08)	0.955
Yes	848 (68)	452 (68.07)	396 (67.92)	
HbA1c ≥5.7%				
No	1087 (77.92)	592 (76.09)	495 (80.23)	0.064
Yes	308 (22.08)	186 (23.91)	122 (19.77)	
SBP ≥130 mmHg and/DBP ≥85 mmHg				
No	1194 (85.59)	660 (84.83)	534 (86.55)	0.365
Yes	201 (14.41)	118 (15.17)	83 (13.45)	
Number of abnormal components				
0	197 (14.12)	125 (16.07)	72 (11.67)	0.079
1	489 (35.05)	283 (36.38)	206 (33.39)	
2	484 (34.7)	257 (33.03)	227 (36.79)	
3	198 (14.19)	100 (12.85)	98 (15.88)	
4	23 (1.65)	11 (1.41)	12 (1.94)	
5	4 (0.29)	2 (0.26)	2 (0.32)	
MetS at baseline^a^				
No	1170 (83.87)	665 (85.48)	505 (81.85)	0.067
Yes	225 (16.13)	113 (14.52)	112 (18.15)	

Values in bold indicate *p*-value ≤0.05.

^a^ MetS according to the presence of ≥3 abnormal cardiometabolic risk components.

MetS, metabolic syndrome.

#### Adiponectin and baseline MetS components in the whole cohort

In the combined cohort, s-ADP showed a significant negative association with WC and a significant positive association with HDL-C in both unadjusted and adjusted models (both *p* < 0.001) (Table 4). In multivariate analyses, s-ADP was significantly negatively associated with WC only when applying an s-ADP x rape-exposure interaction term (β = −0.555 [standard error {SE} 0.148], *p* ≤ 0.001) (Supplementary Table S3).

**Table 4.** Linear Regression Models of the Associations of Baseline Adiponectin with Baseline Metabolic Syndrome Components for the Whole Cohort

Outcome	Model 1			Model 2			Model 3		
	β	SE (β)	*p*	β	SE (β)	*p*	β	SE (β)	*p*
WC	−0.379	0.071	**<0.001**	−0.255	0.072	**<0.001**	−0.26	0.07	**<0.001**
SBP	−0.063	0.044	0.155	0.008	0.037	0.824	0.005	0.037	0.893
DBP	−0.065	0.039	0.096	−0.04	0.034	0.241	−0.04	0.034	0.247
HbA1c	−0.002	0.003	0.506	0.002	0.003	0.519	0.002	0.003	0.511
HDL	0.009	0.002	**<0.001**	0.007	0.002	**<0.001**	0.007	0.002	**<0.001**
Triglycerides	−0.006	0.002	0.007	−0.002	0.002	0.35	−0.002	0.002	0.377

Values in bold indicate *p*-value ≤0.05.

Model 1: Unadjusted.

Model 2: Adjust for baseline metabolic factors (WC, SBP and DBP, HbA1c, HDL, triglycerides).

Model 3: Adjust for baseline metabolic factors (WC, SBP and DBP, HbA1c, HDL, triglycerides) and other covariates (age, HIV, alcohol use, smoking, childhood trauma, lifetime trauma, baseline depression, and baseline PTSD).

#### Leptin, resistin, L/A ratio, and baseline MetS components in the RE group

In the RE group, there were significant positive associations between L/A ratio and SBP, DBP, and triglycerides in the unadjusted models. However, these associations were not retained in the adjusted models (Supplementary Table S4). All three adipokine markers were significantly associated with WC (all *p* < 0.001) in the fully adjusted models. Surprisingly, leptin was positively associated with HDL-C (β = 0.004 [SE 0.002], *p* = 0.046); however, this was not significant after Bonferroni adjustment for multiple testing.

#### Adipokine levels and MetS prevalence

s-ADP was significantly associated with lower MetS prevalence at baseline in the RE group (adjusted odds ratio [aOR]: 0.91, 95% confidence interval [CI]: 0.88–0.95, *p* ≤ 0.001), as well as the whole cohort (aOR: 0.96, 95% CI: 0.93–0.99, *p* = 0.005). In addition, leptin, resistin, and L/A ratio were significantly associated with an increased point prevalence of MetS at baseline in the RE group, after controlling for covariates (all *p* ≤ 0.001) and Bonferroni correction for multiple testing (Table 5).

**Table 5.** Multivariate Logistic Regression Analyses Examining the Associations of Baseline Adipokines with Metabolic Syndrome Prevalence at Baseline

Study population	Adipokine	No. of Cases	Prevalence rates of MetS % (95% CI)	Unadjusted OR (95% CI)	*p*	Multivariable adjusted^b^ OR (95% CI)	*p*
All participants	Adiponectin	225/1395	16.1 (14.3–18.2)	0.96 (0.93–0.99)	**0.004**	0.96 (0.93–0.99)	**0.005**
Rape Exposed							
	Leptin	113/778	14.5 (12.2–17.2)	1.05 (1.03–1.06)	**<0.001**	1.04 (1.03–1.06)	**<0.001**
	Resistin	113/778		1.00 (0.98–1.02)	0.975	1.00 (0.98–1.03)	0.808
	L/A Ratio	113/778		1.36 (1.23–1.51)	**<0.001**	1.35 (1.22–1.50)	**<0.001**
	Adiponectin	113/778		0.91 (0.88–0.95)	**<0.001**	0.92 (0.88–0.95)	**<0.001**

Multivariable models are adjusted for age and HIV status. Values in bold indicate *p*-value ≤0.05. Value in bold indicates significance after adjusting for multiple hypotheses (at α = 0.05/5).

CI, confidence interval; OR, odds ratio.

### Longitudinal analyses

#### Adipokine levels and longitudinal changes in individual MetS components

In multivariable-adjusted linear regression models, baseline s-ADP was not associated with relative change in any of the MetS components in the overall cohort (Supplementary Table S5). Among RE participants specifically, s-ADP was significantly associated with longitudinal change in WC, DBP, HbA1c, and HDL-C in unadjusted models (Supplementary Table S6). After adjusting for confounders, only the association with HDL-C remained significant (β = 0.146 [SE 0.064], *p* = 0.022). When applying a rape interaction term, a significant negative association was shown between s-ADP and relative change in HDL-C (β = −0.01 [SE 0.004], *p* = 0.023) (Supplementary Table S6). However, neither of the associations shown with HDL-C retained significance after applying Bonferroni correction on the resulted *p*-values.

In the RE group, leptin (β = −0.004 [SE 0.002], *p* = 0.012) and resistin (β = −0.003 [SE 0.001], *p* = 0.042) were both significantly negatively associated with longitudinal change in HDL-C before Bonferroni correction was applied (Supplementary Table S7).

In multivariate analysis the results remained similar to our main findings, including no association between baseline s-ADP and longitudinal change in any cardiometabolic component in the overall cohort. Among RE participants, leptin was inversely associated with longitudinal change in HDL-C (β = −0.001 [SE 0.0004], *p* = 0.036) and positively associated with triglycerides (β = 0.003 [SE 0.001], *p* = 0.017). In addition, L/A ratio was shown to be significantly negatively associated with longitudinal change in WC (β = −0.003 [SE 0.001], *p* = 0.016) (Supplementary Table S8).

#### Adipokines and MetS incidence at 12 months

A total of 67 (8.6%) participants (23 RE and 44 RUE) had developed new-onset MetS at 12-month follow-up. Baseline s-ADP, resistin, nor leptin were associated with MetS incidence at 12-month follow-up. On multivariable logistic regression, adjusted for baseline MetS component levels and HIV status, a higher L/A ratio was shown to be associated with a reduced risk of MetS incidence (aOR: 0.58, 95% CI 0.37–0.92, *p* = 0.021) among the RE participants (Supplementary Table S9).

#### Retention

There was a higher retention rate in the RUE group (*n* = 504, 81.6%) compared to the RE group (*n* = 421, 54%) at 12-month follow-up. In both groups, obese individuals were more likely to complete all visits. In the RE group, participants with PTSD at baseline were more likely to be lost to follow-up (*p* = 0.028) (Supplementary Table S1).

## Discussion

To the best of our knowledge, this is the first study to prospectively establish the role of leptin, adiponectin, resistin, and L/A ratio with cardiometabolic outcomes among RE adult females.

Our main findings, in cross-sectional analysis, include a positive association of serum adiponectin with increased HDL-C in both the RE and RUE groups, as has been observed in other cross-sectional studies.^48,49^ Second, and in keeping with our first hypotheses, higher s-ADP and lower leptin and L/A ratio were associated with lower MetS prevalence at baseline. In the RE group specifically, adipokine levels were associated with BMI parameters at baseline. Serum adiponectin and leptin showed reciprocal relationships with increased adiposity, which corresponds with the existing literature.^49,50^ Leptin was positively associated with HDL-C in the RE group, which contrasts with results from other studies that have shown an inverse association between s-ADP and HDL-C.^51^ In total, our results confirm and extend previous cross-sectional observations in other populations to a younger adult female population.^52,53^

We found no longitudinal associations between adiponectin, leptin, or resistin and MetS incidence at 12 months. A higher L/A ratio was shown to be associated with a reduced risk of MetS incidence in RE. However, as no significant associations were shown in the unadjusted model, this is likely a spurious association.^54^ In contrast, previous studies, conducted in older participants over longer periods and with a higher incidence of MetS, found that these adipokines were informative biomarkers for the future risk of MetS.^26,49,55,56^ The latter studies were conducted in aging black South African women over a period of 10 years,^49^ and in middle-aged and elderly male and female Koreans over a 2.4-year follow-up period.^26,56,57^ This suggests a long-term influence of adipokines on the development of MetS, with a greater effect in older individuals and highlights the need for further research to be conducted over longer periods in RE women in South Africa. Adiponectin levels have been shown to increase,^55^ while serum leptin levels decline with aging.^58^

However, the absence of longitudinal associations in this study may be also linked to the influence of HIV-infection, HIV-associated lipodystrophy, and highly active anti-retroviral therapy (HAART) on adipokine secretion in our cohort, which had a high prevalence of HIV infection.^59–61^ Both adiponectin and leptin levels have been shown to be reduced in patients with HAART-related lipodystrophy syndrome^62^; a constellation of a morphological and metabolic changes, including dyslipidemia, insulin resistance, and abnormal fat distribution.^63,64^ Other studies have reported reduced adiponectin levels in HIV-infected patients who were treatment-naive versus those established on HAART.^65^

That adiponectin was significantly inversely associated with longitudinal changes in HDL-C when applying an adiponectin x rape interaction term in the RE group was an unexpected finding and requires further investigation. This contrasts with another longitudinal Korean study that found higher s-ADP to be associated with a reduced risk of incident low HDL-C.^56^ Since low HDL-C is an independent risk factor for cardiovascular diseases of heart attack and stroke,^66^ this association needs to be explored further in other cohorts of women who have been sexually assaulted. Leptin and resistin were inversely associated with longitudinal changes in HDL-C, in keeping with existing literature.^67–69^

In contrast to our third hypothesis, baseline s-ADP levels, as measured on average 12 days post-rape, were comparable between the two groups. This does not accord with two other studies that reported reduced s-ADP levels in the early days following other physical traumas (burns, motor vehicle accidents).^70,71^ Nearly every type of physical trauma mechanism can result in injury to adipose tissue.^72^ Disruption of macro- and micro-barriers (*e.g.*, skin and cell membranes) results in a complex immune response, including the release of various pro-inflammatory mediators such as interleukin-6 and anti-inflammatory mediators such as adiponectin from the injured adipose tissue.^72,73^ In addition, adipose tissue has bidirectional interactions with the HPA-axis.^74^

Any physical or psychological stimuli that disrupts homeostasis result in a stress response, which is mediated by a complex interplay of neuroendocrine and immune mechanisms involving activation of the sympathetic-adreno-medullar (SAM) axis, HPA-axis, and immune system.^75,76^ Post-trauma, catecholamine and inflammatory mediators are released through SAM axis activation, which is thought to dysregulate adipose tissue homeostasis, further aggravating the systemic inflammatory response by adipose tissue itself.^72^ Cortisol, the major HPA-axis hormone, released by the adrenal cortex during the stress response, has been shown to have an inhibitory effect on adiponectin.^77–79^

Therefore, given the complex interplay between the central and peripheral neuroendocrine and inflammatory systems, s-ADP levels are likely to have been impacted by the timing of measurement post-trauma and perhaps also the type of the trauma. For example, for RE individuals, it may be that changes in s-ADP levels only reflect over time where post-traumatic stress reactions are sustained, and aberrant cortisol secretion occurs resulting in HPA axis dysregulation.^11,80,81^ HPA dysfunction has been linked to obesity and MetS.^82,83^

Lastly, in the present study, we found that the observed associations between s-ADP and longitudinal change in cardiometabolic components in the RE group were attenuated after adjusting for mental health (depression, PTSD, lifetime trauma) and risky behavioral factors (alcohol use and tobacco smoking). This is perhaps not surprising considering that sexual violence has well documented adverse impacts on physical and mental health in women, including an increased risk of PTSD and depression^5,24,84,85^ and an increased uptake of risky behaviors such as alcohol abuse and tobacco smoking.^86,87^ However, there is a growing body of literature demonstrating reduced s-ADP levels among individuals with depression and PTSD.^29,88^ Consequently, our study highlights the importance of adjusting for these factors when investigating the associations of adipokine markers with cardiometabolic outcomes among women who have been sexually assaulted or other populations where mental health factors may be prevalent.

### Strengths and limitations

The main strength of this study is that we were able to evaluate cardiometabolic outcomes in relation to three important adipokines cross-sectionally and longitudinally over a period of 1 year in a sizeable subset of RE participants, including a matched RUE cohort. The comprehensive assessments allowed us to control for many potential confounders documented to correlate with both cardiometabolic outcomes and adipokine levels, such as behavioral and mental health risk factors, enhancing the robustness of our findings. Our findings add to the few prior studies that have examined the relation of adipokines with cardiometabolic outcomes longitudinally.

Study limitations include not assessing other potential influences of s-ADP levels such dietary factors and physical activity levels. Nevertheless, other behavioral risk factors of alcohol consumption, which reduces adiponectin and leptin levels,^89,90^ and tobacco smoking were included as covariables. We did not have information regarding medication use (including HAART) and, therefore, cannot exclude the effect of medication on cardiometabolic parameters. We evaluated serum adipokine levels at baseline only, which may not adequately capture their biological activity or variability over time. Due to costs, we were not able to obtain leptin and resistin levels for the RUE group. The absence of fasting blood samples may affect the levels of adipokines and other cardiometabolic variables, particularly triglyceride and glucose levels. Diabetes and dysglycemia were not diagnosed on oral glucose tolerance tests but HbA1c is considered a feasible alternative. There is increasing recognition of the clinical utility and applicability of nonfasting samples for determining lipid profiles.^91,92^

There was a high attrition rate in the RE group, and those lost to follow-up may have experienced an improvement or deterioration in their cardiometabolic profiles. The sample size of incident MetS among RE was small (*N* = 44), limiting our statistical power to assess associations in this group. Finally, the results of this study cannot be generalized to other populations, and it will be necessary to replicate study findings in other large well characterized study populations.

These limitations notwithstanding, our data provide tentative insights into the potential for novel biomarkers to predict the development of CMDs alone and in combination (as exemplified by MetS). The range of associations shown for these adipokines with adiposity, lipid-profile and MetS prevalence, supports their possible pathophysiological role in RE individuals specifically. There is increasing recognition of the significant health care disparities and gaps that persist in the care and outcomes of women with CMD.^93,94^ Adipokines may have potential clinical utility to identify traumatized woman at risk for CMD early; however, the current high cost of testing may limit feasibility in practice.

Future research investigating associations between adipokine markers and CMD must consider other potential confounders (dietary habits, sedentary behavior, use of and adherence to chronic medication), longer period of follow-up, inclusion of older women who are at higher risk of CMD, and repeat measurements of adipokine levels to address research gaps.

## Conclusions

In conclusion, our results suggest a role for adipokines in the pathophysiology of MetS in RE women. Additional research is needed to further explore the physiologic pathways whereby adiponectin, leptin, resistin, and L/A ratio contribute to cardiometabolic dysregulation and increase the risk of incident MetS following sexual assault. If confirmed, measuring adipokines following trauma could provide a picture of the state of cardiometabolic health over time and inform timely interventions.

## Supplementary Material

Supplemental data

## Data Availability

The data that support the findings of this study are being used to address additional study aims and cannot be shared publicly at this stage. The study collaborative team will review individual requests to access data.

## Abbreviations Used

aOR: adjusted odds ratio
AUDIT-C: Alcohol Use Disorders Identification Test-C
BMI: body mass index
BP: blood pressure
CES-D: Centre for Epidemiologic Studies Depression Scale
CI: confidence interval
CMD: cardiometabolic disease
CTQ-SF: Childhood Trauma Questionnaire-Short Form
DBP: diastolic blood pressure
ELISA: enzyme-linked immunosorbent assay
HAART: highly active anti-retroviral therapy
HbA1c: glycated hemoglobin
HC: hip circumference
HDL-C: high-density lipoprotein cholesterol
HPA: hypothalamic pituitary adrenal
IQR: interquartile range
L/A: leptin/adiponectin
LDL-C: low-density lipoprotein cholesterol
LEC: life events checklist
MetS: metabolic syndrome
OR: odds ratio
PTSD: posttraumatic stress disorder
RE: rape-exposed
RICE: Rape Impact Cohort Evaluation
RUE: rape-unexposed
s-ADP: serum adiponectin
SAM: sympathetic-adreno-medullar
SAMRC: South African Medical Research Council Ethics Committee
SBP: systolic blood pressure
SD: standard deviation
SE: standard error
WC: waist circumference
WHR: waist-to-hip ratio
