# Supplementary material for: Prospective Association of Circulating Adipokines with Cardiometabolic Risk Profile Among Women: The Rape Impact Cohort Evaluation Study
Source: Womens Health Rep (New Rochelle). 2022 Oct 7;3(1):820–33. doi: 10.1089/whr.2022.0069 (PMC9629977; doi:10.1089/whr.2022.0069)
Supplement: Supplemental data [file Supp_Data.docx]

**Supplementary files**

**Prospective association of circulating adipokines with cardiometabolic risk profile among women : The Rape Impact Cohort Evaluation (RICE) study.**

Vuong Eileen^1,2*^, Peer Nasheeta^3,^ Chirwa Esnat^4,8,^ Mhlongo Shibe^4^, Lombard Carl^5^, Hemmings Sian^1,6^, Kengne Andre Pascal^3^, Abrahams Naeemah^4,7^, Seedat Soraya^1,2^

^1^South African Research Chairs Initiative (SARChI), PTSD program, Department of Psychiatry, Stellenbosch University, South Africa.

^2^Department of Psychiatry, Stellenbosch University, Stellenbosch, South Africa

^3^Non-Communicable Diseases Research Unit, South African Medical Research Council, and Department of Medicine, University of Cape Town

^4^Gender and Health Research Unit, South African Medical Research Council, Tygerberg, South Africa

^5^Biostatitistics Unit, South African Medical Research Council

^6^Division of Molecular Biology and Human Genetics, Stellenbosch University, South Africa.

^7^School of Public Health and Family Medicine: Faculty of Health Sciences, University of Cape Town.

^8^School of Public Health, Faculty of Health Sciences, University of Witwatersrand.

Correspondence:

Dr Eileen Vuong

Department of Psychiatry

Stellenbosch University

PO Box 241, Cape Town 8000

South Africa

Phone: +27219389780

Fax: +27214488158

Email: [eileenthomas@sun.ac.za](mailto:eileenthomas@sun.ac.za)

**S1:** **Baseline demographic and clinical differences of study participants by retention and follow-up status**

|  | **Rape unexposed (N=617)** | | | **Rape exposed (N=778)** | | |
| --- | --- | --- | --- | --- | --- | --- |
| **Variables** | **Available at 12-month visit (n=504)** | **Missed 12-month visit (n=113)** |  | **Available at 12-month visit (n=421)** | **Missed 12-month visit (n=357)** |  |
|  | **n(%)/mean(sd)** | **n(%)/mean(sd)** | **p-value** | **n(%)/mean(sd)** | **n(%)/mean(sd)** | **p-value** |
| **Socio-demographics** |  |  |  |  |  |  |
| Age : mean (SD) | 25.8(5.4) | 25.1(5.3) | 0.212 | 25(5.4) | 24.8(5.2) | 0.612 |
| Education | 305(60.52) | 70(61.95) | 0.778 | 238(56.53) | 208(58.26) | 0.627 |
| Employment | 70(13.89) | 21(18.58) | 0.203 | 101(23.99) | 102(28.57) | 0.147 |
| **Mental Health** |  |  |  |  |  |  |
| Depression^a^ | 160(31.75) | 33(29.2) | 0.598 | 379(90.02) | 326(91.32) | 0.538 |
| PTSD^b^ | 19(3.77) | 4(3.54) | 0.907 | 63(14.96) | 75(21.01) | 0.028 |
| Childhood trauma (CTQ): mean (SD) | 15.9(2.5) | 15.6(2.7) | 0.258 | 16.7(3.8) | 16.5(3.6) | 0.382 |
| Lifetime trauma (LEC): mean (SD) | 1.2(1.5) | 1.2(1.6) | 0.922 | 2.1(1.8) | 1.9(1.8) | 0.259 |
| **Behavioural** |  |  |  |  |  |  |
| Harmful alcohol use^c^ | 123(24.4) | 18(15.93) | 0.052 | 137(32.54) | 102(28.57) | 0.232 |
| Smoker^d^ | 51(10.12) | 8(7.08) | 0.321 | 64(15.2) | 52(14.57) | 0.804 |
| **Clinical measurements** |  |  |  |  |  |  |
| Abdominal obesity,%^e^ | 209(41.47) | 35(30.97) | **0.039** | 115(27.32) | 69(19.33) | **0.009** |
| Abnormal BP, %^f^ | 68(13.49) | 15(13.27) | 0.951 | 63(14.96) | 53(14.85) | 0.963 |
| **Biochemical measurements** |  |  |  |  |  |  |
| Dysglycemia^g^ | 96(19.05) | 26(23.01) | 0.339 | 99(23.52) | 87(24.37) | 0.781 |
| Hypertriglyceridemia^h^ | 18(3.77) | 7(6.67) | 0.184 | 13(3.62) | 11(3.61) | 0.992 |
| Reduced HDL-C^i^ | 324(67.78) | 72(68.57) | 0.875 | 246(68.52) | 206(67.54) | 0.787 |
| **Adipokines** | () | () |  | () | () |  |
| Adiponectin (µg/mL): mean (SD) | 13(4.2) | 12.3(4.6) | 0.096 | 12.9(5.9) | 12.6(5.7) | 0.616 |
| Leptin (ng/mL): mean (SD) |  |  |  | 13.3(12) | 13(9.9) | 0.688 |
| Resistin (ng/mL): mean (SD) |  |  |  | 14.4(9.7) | 13.7(8.4) | 0.291 |
| Leptin/adiponectin ratio: mean (SD) |  |  |  | 1.5(2.1) | 1.4(1.7) | 0.676 |
| **Clinical conditions** |  |  |  |  |  |  |
| HIV positive | 201(39.88) | 41(36.28) | 0.479 | 201(47.74) | 168(47.06) | 0.849 |

Values for continuous variables are presented as mean ± SD or as median & IQR; ^a^Depression based on CESD ≥ 16; ^b^PTSD according to Mini Neuropsychiatric interview, ^c^Harmful alcohol use according to AUDIT-C ≥ 3; ^d^Smoker includes daily and occasional smokers; ^e^Abdominal obesity: Body mass index ≥30kg/m^2^; ^f^Abnormal blood pressure: systolic blood pressure (BP) ≥130 mmHg or diastolic BP ≥85 mmHg or self-reported hypertension; ^g^Dysglycaemia: Hb1A_c_ ≥ 5.7 mmol/L or self-reported diabetes; ^h^Hypetriglyceridemia: Triglycerides ≥1.7mmol/L; ^I^Low HDL_C: HDL-C≤1.3 mmol/L. Abbreviations: CTQ, Childhood trauma questionnaire; LEC, Life events checklist; HDL-C, high-density lipoprotein cholesterol; LDL-C, low-density lipoprotein cholesterol. Values in bold indicate p≥ 0.05

**S2. Power calculation**

**Tests for Two Proportions**

**Numeric Results for Testing Two Proportions using the Z-Test with Unpooled Variance ─────────────**

Hypotheses: H0: P1 - P2 = 0 vs. H1: P1 - P2 ≠ 0

**Target Actual Diff**

**Power* N1 N2 N %N1 %N1 P1 P2 D1 Alpha**

0,43613 365 547 912 40 40,02193 0,140 0,1 0,040 0,05

0,59708 365 547 912 40 40,02193 0,150 0,1 0,050 0,05

0,73876 365 547 912 40 40,02193 0,160 0,1 0,060 0,05

0,79730 365 547 912 40 40,02193 0,165 0,1 0,065 0,05

0,84657 365 547 912 40 40,02193 0,170 0,1 0,070 0,05

0,91835 365 547 912 40 40,02193 0,180 0,1 0,080 0,05

- Power was computed using the normal approximation method.
- Prevalence of MetS used to calculate the power was 20%.

**Report Definitions**

Power is the probability of rejecting a false null hypothesis. N1 and N2 are the number of items sampled from each population. N is the total sample size, N1 + N2. Target %N1 is the desired percent (or precents) of the total sample size to be allocated to Group 1, as entered in the procedure.

Actual %N1 is the percent in N1 obtained. Because N1 and N2 are discrete, this value is sometimes slightly different than the target %N1.

P1 is the proportion for Group 1 at which power and sample size calculations are made. This is the treatment or experimental group. P2 is the proportion for Group 2. This is the standard, reference, or control group. D1 is the difference P1 - P2 assumed for power and sample size calculations.

Alpha is the probability of rejecting a true null hypothesis.

**Summary Statements ─────────────────────────────────────────────────────────**

Group sample sizes of 365 in group 1 and 547 in group 2 achieve 43,613% power to detect a difference between the group proportions of 0,04. The proportion in group 1 (the treatment group) is assumed to be 0,1 under the null hypothesis and 0,14 under the alternative hypothesis. The proportion in group 2 (the control group) is 0,1. The test statistic used is the two-sided Z-Test with unpooled variance. The significance level of the test is 0,05.

**
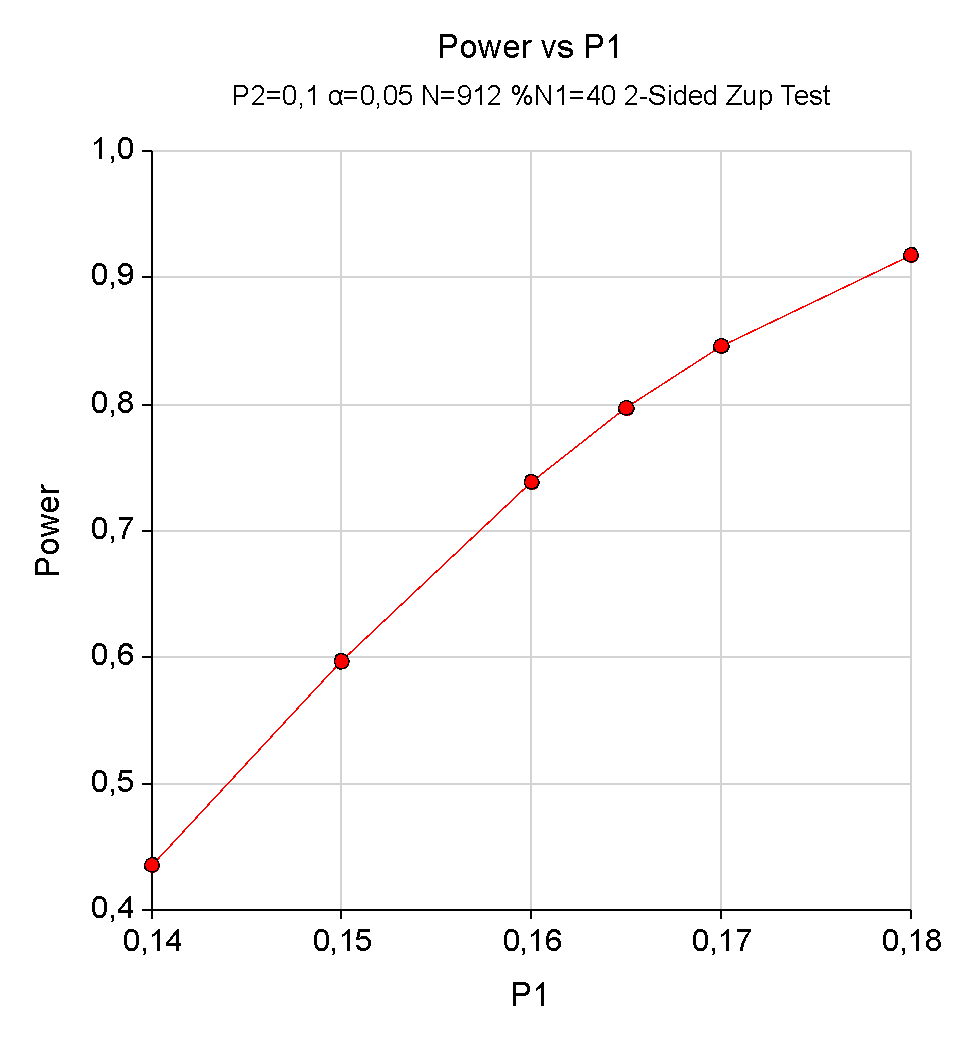
**

**References**

Chow, S.C., Shao, J., and Wang, H. 2008. Sample Size Calculations in Clinical Research, Second Edition. Chapman & Hall/CRC. Boca Raton, Florida.

D'Agostino, R.B., Chase, W., and Belanger, A. 1988. 'The Appropriateness of Some Common Procedures for Testing the Equality of Two Independent Binomial Populations', The American Statistician, August 1988, Volume 42 Number 3, pages 198-202.

Fleiss, J. L., Levin, B., and Paik, M.C. 2003. Statistical Methods for Rates and Proportions. Third Edition. John Wiley & Sons. New York.

Lachin, John M. 2000. Biostatistical Methods. John Wiley & Sons. New York.

Machin, D., Campbell, M., Fayers, P., and Pinol, A. 1997. Sample Size Tables for Clinical Studies, 2nd Edition. Blackwell Science. Malden, Mass.

Ryan, Thomas P. 2013. Sample Size Determination and Power. John Wiley & Sons. Hoboken, New Jersey.

**S3.** **Multivariate analysis of the relationship between baseline adiponectin and baseline levels of MetS components**

| **Outcome** |  | **Model 1** | | | **Model 2** | | | **Model 3** | | |
| --- | --- | --- | --- | --- | --- | --- | --- | --- | --- | --- |
| **Baseline** | **Main exposure** | **β** | **S.E(β)** | **p-value** | **β** | **S.E(β)** | **p-value** | **β** | **S.E(β)** | **p-value** |
| WC | Adiponectin | 0.16 | 0.127 | 0.209 | 0.147 | 0.125 | 0.238 | 0.116 | 0.122 | 0.34 |
|  | Rape exposure | 5.128 | 2.085 | **0.014** | 2.915 | 2.085 | 0.162 | 4.221 | 2.138 | **0.049** |
|  | Adiponectin x rape exposure | -0.78 | 0.152 | **<0.001^*^** | -0.596 | 0.151 | **<0.001^*^** | -0.555 | 0.148 | **<0.001^*^** |
| SBP | Adiponectin | 0.031 | 0.081 | 0.698 | -0.03 | 0.066 | 0.651 | -0.038 | 0.065 | 0.555 |
|  | Rape exposure | 0.011 | 1.323 | 0.993 | -0.773 | 1.097 | 0.481 | -0.869 | 1.139 | 0.446 |
|  | Adiponectin x rape exposure | -0.138 | 0.096 | 0.151 | 0.056 | 0.08 | 0.481 | 0.064 | 0.079 | 0.416 |
| DBP | Adiponectin | 0.083 | 0.071 | 0.242 | 0.042 | 0.059 | 0.482 | 0.044 | 0.059 | 0.462 |
|  | Rape exposure | 0.852 | 1.159 | 0.462 | 0.513 | 0.991 | 0.605 | 0.616 | 1.044 | 0.556 |
|  | Adiponectin x rape exposure | -0.215 | 0.084 | **0.011** | -0.124 | 0.072 | 0.087 | -0.124 | 0.073 | 0.088 |
| HbA1c | Adiponectin | 0.009 | 0.005 | 0.094 | 0.01 | 0.005 | **0.035** | 0.01 | 0.005 | **0.042** |
|  | Rape exposure | 0.26 | 0.084 | **0.002^*^** | 0.215 | 0.083 | **0.01** | 0.191 | 0.088 | **0.03** |
|  | Adiponectin x rape exposure | -0.015 | 0.006 | **0.016** | -0.013 | 0.006 | **0.036** | -0.012 | 0.006 | **0.043** |
| HDL-C | Adiponectin | 0.003 | 0.003 | 0.413 | 0.003 | 0.003 | 0.305 | 0.003 | 0.003 | 0.369 |
|  | Rape exposure | -0.143 | 0.053 | **0.007^*^** | -0.103 | 0.052 | **0.047** | -0.104 | 0.054 | 0.054 |
|  | Adiponectin x rape exposure | 0.01 | 0.004 | **0.012** | 0.006 | 0.004 | 0.134 | 0.006 | 0.004 | 0.097 |
| Triglycerides | Adiponectin | -0.002 | 0.004 | 0.656 | -0.003 | 0.004 | 0.488 | -0.004 | 0.004 | 0.358 |
|  | Rape exposure | 0.134 | 0.069 | 0.052 | 0.058 | 0.066 | 0.383 | 0.038 | 0.069 | 0.582 |
|  | Adiponectin x rape exposure | -0.007 | 0.005 | 0.196 | 0.001 | 0.005 | 0.825 | 0.002 | 0.005 | 0.612 |
| **Model 1: unadjusted** | | | | | | | | | | |
| **Model 2: adjust for baseline metabolic factors and HIV** | | | | | | | | | | |
| **Model 3: adjust for baseline metabolic factors, HIV, and other covariates; age, HIV, alcohol use, smoking, childhood trauma, lifetime trauma, depression, and PTSD** | | | | | | | | | | |

Values in bold indicate p-value ≤ 0.05 (unadjusted for multiple testing). Significant p-value at α*=0.008 (Bonferroni adjustment for multiple testing (6 Mets outcomes)) α*= α/no of hypotheses, where α=0.05; β, beta coefficient; S.E(β), standard error(β).

**S4. Linear regression models of the associations of leptin, resistin and leptin/adiponectin ratio with MetS components at baseline**

|  |  |  | **Model 1** |  |  |  | **Model 2** |  |  |  | **Model 3** |  |  |
| --- | --- | --- | --- | --- | --- | --- | --- | --- | --- | --- | --- | --- | --- |
| Outcome | Exposure | **Coef.** | **S.E** | **p-value** |  | **Coef.** | **S.E** | **p-value** |  | **Coef.** | **S.E** | **p-value** |  |
| Waist circumference | Leptin | 0.645 | 0.044 | **<0.001^*^** |  | 0.694 | 0.052 | **<0.001^*^** |  | 0.685 | 0.052 | **<0.001^*^** |  |
| Systolic blood pressure |  | 0.096 | 0.033 | **0.003^*^** |  | -0.033 | 0.041 | 0.417 |  | -0.039 | 0.041 | 0.339 |  |
| Diastolic blood pressure |  | 0.074 | 0.029 | **0.010** |  | -0.005 | 0.039 | 0.89 |  | 0.01 | 0.04 | 0.8 |  |
| Glycated haemoglobin |  | 0.002 | 0.002 | 0.360 |  | -0.001 | 0.003 | 0.615 |  | -0.002 | 0.003 | 0.579 |  |
| High density lipoprotein |  | -0.003 | 0.002 | 0.060 |  | 0.004 | 0.002 | 0.085 |  | 0.004 | 0.002 | **0.046** |  |
| Triglycerides |  | 0.003 | 0.002 | 0.152 |  | -0.002 | 0.003 | 0.391 |  | -0.001 | 0.003 | 0.607 |  |
|  |  |  |  |  |  |  |  |  |  |  |  |  |  |
| Waist circumference | Resistin | 0.182 | 0.067 | **<0.001^*^** |  | 0.204 | 0.065 | **0.002^*^** |  | 0.234 | 0.065 | **<0.001^*^** |  |
| Systolic blood pressure |  | -0.021 | 0.041 | 0.610 |  | -0.029 | 0.035 | 0.406 |  | -0.015 | 0.035 | 0.664 |  |
| Diastolic blood pressure |  | 0.003 | 0.036 | 0.920 |  | 0.018 | 0.033 | 0.591 |  | 0.01 | 0.034 | 0.767 |  |
| Glycated haemoglobin |  | -0.003 | 0.002 | 0.190 |  | -0.002 | 0.002 | 0.4 |  | -0.001 | 0.002 | 0.577 |  |
| High density lipoprotein |  | 0 | 0.002 | 0.95 |  | 0.002 | 0.002 | 0.362 |  | 0.001 | 0.002 | 0.527 |  |
| Triglycerides |  | 0 | 0.002 | 0.952 |  | -0.001 | 0.002 | 0.534 |  | -0.002 | 0.002 | 0.327 |  |
|  |  |  |  |  |  |  |  |  |  |  |  |  |  |
| Waist circumference | L/A | 3.566 | 0.263 | **<0.001^*^** |  | 4.064 | 0.341 | **<0.001^*^** |  | 3.967 | 0.339 | **<0.001^*^** |  |
| Systolic blood pressure |  | 0.746 | 0.19 | **<0.001^*^** |  | -0.134 | 0.252 | 0.594 |  | -0.123 | 0.246 | 0.618 |  |
| Diastolic blood pressure |  | 0.613 | 0.168 | **<0.001^*^** |  | 0.205 | 0.239 | 0.392 |  | 0.239 | 0.24 | 0.32 |  |
| Glycated haemoglobin |  | 0.015 | 0.01 | 0.130 |  | 0.002 | 0.017 | 0.9 |  | 0.002 | 0.017 | 0.905 |  |
| High density lipoprotein |  | -0.038 | 0.012 | **0.001^*^** |  | -0.001 | 0.013 | 0.966 |  | 0.002 | 0.014 | 0.884 |  |
| Triglycerides |  | 0.032 | 0.014 | **0.020** |  | 0.001 | 0.017 | 0.945 |  | 0.004 | 0.017 | 0.796 |  |
| Model 1: unadjusted  Model 2: adjust for baseline metabolic factors and HIV  Model 3: adjust for baseline metabolic factors, HIV, and other covariates (age, alcohol use, smoking, childhood trauma, lifetime trauma, depression, and PTSD) | | | | | | | | | | | | | |

Values in bold indicate p-value ≤ 0.05 (unadjusted for multiple testing). * Significant p-value at α*=0.008 (Bonferroni adjustment for multiple testing (6 Mets outcomes for each adipokine marker)) α*= α/no of hypotheses, where α=0.05; β, beta coefficient; S.E(β), standard error(β).

**S5.** **Linear regression analyses examining the associations between baseline adiponectin and relative changes in MetS component levels at 12-month follow-up**

| **Outcome** |  | **Model 1** | | | **Model 2** | | | **Model 3** | | | **Model 4** | | |
| --- | --- | --- | --- | --- | --- | --- | --- | --- | --- | --- | --- | --- | --- |
| **Relative change in:** | **Main exposure** | **β** | **S.E(β)** | **p-value** | **β** | **S.E(β)** | **p-value** | **β** | **S.E(β)** | **p-value** | **β** | **S.E(β)** | **p-value** |
| Waist circumference | Adiponectin | -0.001 | 0.001 | 0.238 | -0.000 | 0.001 | 0.255 | -0.001 | 0.001 | 0.208 | -0.001 | 0.001 | 0.234 |
|  | Rape exposure |  |  |  | 0.014 | 0.005 | **0.007^*^** | 0.012 | 0.005 | **0.03** | 0.010 | 0.007 | 0.142 |
| Systolic blood pressure | Adiponectin | 0.0003 | 0.001 | 0.577 | 0.400 | 0.001 | 0.588 | -0.001 | 0.001 | 0.428 | -0.0004 | 0.001 | 0.526 |
|  | Rape exposure |  |  |  | 0.005 | 0.006 | 0.357 | -0.005 | 0.006 | 0.4 | -0.006 | 0.007 | 0.463 |
| Diastolic blood pressure | Adiponectin | -0.001 | 0.001 | 0.279 | 0.001 | 0.001 | 0.299 | -0.001 | 0.001 | 0.411 | -0.001 | 0.001 | 0.421 |
|  | Rape exposure |  |  |  | 0.024 | 0.008 | **0.005^*^** | 0.024 | 0.009 | **0.007^*^** | 0.021 | 0.011 | 0.065 |
| Glycated haemoglobin | Adiponectin | -0.002 | 0.001 | 0.147 | 0.002 | 0.001 | 0.134 | -0.002 | 0.001 | 0.171 | -0.002 | 0.001 | 0.15 |
|  | Rape exposure |  |  |  | -0.023 | 0.011 | **0.032** | -0.019 | 0.012 | 0.111 | -0.009 | 0.015 | 0.576 |
| High density lipoprotein | Adiponectin | -0.003 | 0.002 | 0.120 | 0.003 | 0.002 | 0.117 | -0.003 | 0.002 | 0.161 | -0.003 | 0.002 | 0.194 |
|  | Rape exposure |  |  |  | 0.069 | 0.022 | **0.002^*^** | 0.054 | 0.022 | **0.016** | 0.016 | 0.028 | 0.558 |
| Triglycerides | Adiponectin | 0.000 | 0.004 | 0.994 | 0.000 | 0.004 | 0.994 | -0.002 | 0.004 | 0.647 | -0.001 | 0.004 | 0.779 |
|  | Rape exposure |  |  |  | -0.034 | 0.041 | 0.404 | -0.046 | 0.042 | 0.278 | -0.094 | 0.052 | 0.071 |
| Model 1: Unadjusted | | | | | | | | | | | | | |
| Model 2: Adjusted for rape exposure | | | | | | | | | | | | | |
| Model 3: Adjusted for rape exposure, baseline MetS and HIV | | | | | | | | | | | | | |
| Model 4: Adjusted for rape exposure, baseline MetS, HIV and other covariates (age, alcohol use, smoking, lifetime trauma, depression, and PTSD) | | | | | | | | | | | | | |

Values in bold indicate p-value ≤ 0.05 (unadjusted for multiple testing). * Significant p-value at α*=0.008 (Bonferroni adjustment for multiple testing (6 Mets outcomes)) α*= α/no of hypotheses, where α=0.05; β, beta coefficient; S.E(β), standard error(β).

**S6. Linear regression analysis examining associations between adiponectin and relative changes in MetS component levels at 12-month follow-up** **with rape interaction term.**

| **Outcome** |  | **Model 1** | | | **Model 2** | | | **Model 3** | | | **Model 4** | | |
| --- | --- | --- | --- | --- | --- | --- | --- | --- | --- | --- | --- | --- | --- |
| **Relative change in:** | **Main exposure** | **β** | **S.E(β)** | **p-value** | **β** | **S.E(β)** | **p-value** | **β** | **S.E(β)** | **p-value** | **β** | **S.E(β)** | **p-value** |
| WC | Adiponectin | -0.001 | 0.001 | 0.238 | 0 | 0.001 | 0.665 | -0.001 | 0.001 | 0.493 | -0.001 | 0.001 | 0.527 |
|  | Rape exposure |  |  |  | 0.018 | 0.014 | 0.199 | 0.014 | 0.015 | 0.357 | 0.012 | 0.016 | 0.431 |
|  | Adiponectin #rape exposure |  |  |  | 0 | 0.001 | 0.735 | 0 | 0.001 | 0.883 | 0 | 0.001 | 0.87 |
| SBP | Adiponectin | 0 | 0.001 | 0.577 | -0.001 | 0.001 | 0.36 | 0 | 0.001 | 0.678 | 0 | 0.001 | 0.709 |
|  | Rape exposure |  |  |  | -0.006 | 0.016 | 0.718 | -0.003 | 0.016 | 0.844 | -0.005 | 0.017 | 0.779 |
|  | Adiponectin #rape exposure |  |  |  | 0.001 | 0.001 | 0.461 | 0 | 0.001 | 0.906 | 0 | 0.001 | 0.966 |
| DBP | Adiponectin | -0.001 | 0.001 | 0.279 | -0.003 | 0.001 | **0.017** | -0.002 | 0.001 | 0.102 | -0.002 | 0.001 | 0.102 |
|  | Rape exposure |  |  |  | -0.026 | 0.024 | 0.28 | -0.009 | 0.024 | 0.714 | -0.013 | 0.025 | 0.61 |
|  | Adiponectin #rape exposure |  |  |  | 0.004 | 0.002 | **0.026** | 0.003 | 0.002 | 0.149 | 0.003 | 0.002 | 0.144 |
| HbA1c | Adiponectin | -0.002 | 0.001 | 0.147 | -0.003 | 0.002 | 0.052 | -0.003 | 0.002 | 0.068 | -0.003 | 0.002 | 0.071 |
|  | Rape exposure |  |  |  | -0.059 | 0.03 | **0.048** | -0.058 | 0.033 | 0.083 | -0.045 | 0.035 | 0.2 |
|  | Adiponectin #rape exposure |  |  |  | 0.003 | 0.002 | 0.194 | 0.003 | 0.002 | 0.215 | 0.003 | 0.002 | 0.249 |
| HDL-C | Adiponectin | -0.003 | 0.002 | 0.12 | 0.003 | 0.003 | 0.462 | 0.003 | 0.003 | 0.384 | 0.003 | 0.003 | 0.359 |
|  | Rape exposure |  |  |  | 0.196 | 0.062 | **0.002^*^** | 0.183 | 0.061 | **0.003^*^** | 0.146 | 0.064 | **0.022** |
|  | Adiponectin #rape exposure |  |  |  | -0.01 | 0.004 | **0.027** | -0.01 | 0.004 | **0.023** | -0.01 | 0.004 | **0.023** |
| Triglycerides | Adiponectin | 0 | 0.004 | 0.994 | -0.009 | 0.006 | 0.17 | -0.009 | 0.006 | 0.139 | -0.009 | 0.006 | 0.179 |
|  | Rape exposure |  |  |  | -0.223 | 0.114 | 0.051 | -0.211 | 0.115 | 0.067 | -0.259 | 0.12 | 0.032 |
|  | Adiponectin #rape exposure |  |  |  | 0.015 | 0.008 | 0.077 | 0.013 | 0.008 | 0.123 | 0.013 | 0.008 | 0.129 |
| Model 1: Unadjusted; Model 2: Adjusted for rape exposure; Model 3: Adjusted for rape exposure, baseline MetS and HIV; Model 4: Adjusted for rape exposure, baseline MetS, HIV and other covariates (age, alcohol use, smoking, lifetime trauma, depression, and PTSD) | | | | | | | | | | | | | |

Values in bold indicate p-value ≤ 0.05 (unadjusted for multiple testing). * Significant p-value at α*=0.008 (Bonferroni adjustment for multiple testing (6 Mets outcomes)) α*= α/no of hypotheses, where α=0.05; β, beta coefficient; S.E(β), standard error(β).

**S7. Linear regression models of the associations of leptin, resistin and leptin/adiponectin ratio with relative changes in MetS components at 12-month follow-up**

|  |  | **Model 1** | | |  | **Model 2** | | | |  | | **Model 3** | | |  |
| --- | --- | --- | --- | --- | --- | --- | --- | --- | --- | --- | --- | --- | --- | --- | --- |
| **Relative change in:** | **Main exposure** | **β** | **S.E(β)** | **p-value** |  | **Β** | **S.E(β)** | **p-value** |  | | **β** | | **S.E(β)** | **p-value** |  |
| WC | Leptin | -0.0004 | 0.0003 | 0.156 |  | -0.001 | 0.0003 | 0.126 |  | | -0.001 | | 0.0004 | 0.237 |  |
| SBP |  | 0.0003 | 0.0004 | 0.338 |  | 0.001 | 0.001 | 0.121 |  | | 0.001 | | 0.0005 | 0.06 |  |
| DBP |  | 0.0003 | 0.001 | 0.547 |  | 0.001 | 0.001 | 0.35 |  | | 0.001 | | 0.001 | 0.321 |  |
| HbA1c |  | 0.0002 | 0.0003 | 0.468 |  | 0.001 | 0.0003 | 0.187 |  | | 0.001 | | 0.001 | 0.131 |  |
| HDL-C |  | -0.003 | 0.001 | **0.02** |  | -0.004 | 0.002 | **0.018** |  | | -0.004 | | 0.002 | **0.012** |  |
| Triglycerides |  | 0.003 | 0.002 | 0.152 |  | -0.003 | 0.004 | 0.415 |  | | -0.003 | | 0.004 | 0.4 |  |
|  |  |  |  |  |  |  |  |  |  | |  | |  |  |  |
| WC | Resistin | -0.0001 | 0.0004 | 0.785 |  | -0.0001 | 0.0004 | 0.952 |  | | -0.0002 | | 0.0004 | 0.602 |  |
| SBP |  | 0.001 | 0.0004 | 0.212 |  | 0.001 | 0.0004 | 0.075 |  | | 0.001 | | 0.0004 | 0.122 |  |
| DBP |  | 0.001 | 0.001 | 0.075 |  | 0.001 | 0.001 | 0.139 |  | | 0.001 | | 0.001 | 0.14 |  |
| HbA1c |  | 0.001 | 0.004 | **0.025** |  | 0.001 | 0.0002 | 0.052 |  | | 0.001 | | 0.0004 | 0.153 |  |
| HDL-C |  | -0.003 | 0.001 | **0.012** |  | -0.003 | 0.001 | **0.014** |  | | -0.003 | | 0.001 | **0.042** |  |
| Triglycerides |  | -0.006 | 0.003 | 0.078 |  | -0.004 | 0.003 | 0.225 |  | | -0.005 | | 0.003 | 0.128 |  |
|  |  |  |  |  |  |  |  |  |  | |  | |  |  |  |
| WC | L/A | -0.001 | 0.002 | 0.415 |  | -0.002 | 0.003 | 0.326 |  | | -0.002 | | 0.003 | 0.447 |  |
| SBP |  | -0.0001 | 0.002 | 0.956 |  | 0.003 | 0.003 | 0.341 |  | | 0.003 | | 0.003 | 0.288 |  |
| DBP |  | -0.002 | 0.003 | 0.592 |  | 0.001 | 0.005 | 0.85 |  | | 0.001 | | 0.005 | 0.825 |  |
| HbA1c |  | 0.002 | 0.002 | 0.198 |  | 0.006 | 0.003 | 0.059 |  | | 0.006 | | 0.003 | 0.056 |  |
| HDL-C |  | -0.004 | 0.008 | 0.598 |  | -0.004 | 0.01 | 0.723 |  | | -0.004 | | 0.01 | 0.716 |  |
| Triglycerides |  | -0.029 | 0.02 | 0.145 |  | -0.014 | 0.024 | 0.558 |  | | -0.014 | | 0.024 | 0.563 |  |
| Model 1: Unadjusted. Model 2: Adjusted for other baseline MetS and HIV. Model 3: Adjusted for baseline MetS and other covariates (age, HIV, alcohol use, smoking, lifetime trauma, depression, and PTSD). | | | | | | | | | | | | | | |  |
| L/A: leptin/adiponectin ratio; WC, waist circumference; SBP, systolic blood pressure; DBP, diastolic blood pressure; HbA1c, Glycated haemoglobin; HDL, high density lipoprotein | | | | | | | | | | | | | | |  |

Values in bold indicate p-value ≤ 0.05 (unadjusted for multiple testing). * Significant p-value at α*=0.008 (Bonferroni adjustment for multiple testing (6 Mets outcomes for each adipokine marker)) α*= α/no of hypotheses, where α=0.05; β, beta coefficient; S.E(β), standard error(β).

**S8. Multivariate analysis of association between adipokines and relative changes in MetS components at 12-month follow-up**

|  |  | **Model 1** | | | **Model 2** | | | **Model 3** | | |
| --- | --- | --- | --- | --- | --- | --- | --- | --- | --- | --- |
|  | **Relative change in:** | **β** | **S.E(β)** | **p-value** | **β** | **S.E(β)** | **p-value** | **β** | **S.E(β)** | **p-value** |
| Adiponectin | WC | 0.001 | 0.001 | 0.294 | 0.001 | 0.001 | 0.294 | 0.001 | 0.001 | 0.342 |
|  | SBP | -0.001 | 0.001 | 0.358 | -0.001 | 0.001 | 0.356 | -0.001 | 0.001 | 0.347 |
|  | DBP | -0.0003 | 0.001 | 0.654 | -0.0003 | 0.001 | 0.654 | -0.0003 | 0.001 | 0.620 |
|  | HbA1c | -0.001 | 0.001 | 0.362 | -0.001 | 0.001 | 0.359 | -0.001 | 0.001 | 0.362 |
|  | HDL-C | -0.002 | 0.001 | 0.14 | -0.002 | 0.001 | 0.14 | -0.002 | 0.001 | 0.118 |
|  | Triglycerides | -0.003 | 0.002 | 0.117 | -0.003 | 0.002 | 0.115 | -0.003 | 0.002 | 0.158 |
| Leptin | WC | -0.001 | 0.0004 | 0.14 |  |  |  | -0.001 | 0.0004 | 0.196 |
|  | SBP | -0.001 | 0.0004 | 0.109 |  |  |  | -0.001 | 0.0004 | 0.242 |
|  | DBP | 0.001 | 0.0004 | 0.244 |  |  |  | 0.001 | 0.0004 | 0.152 |
|  | HbA1c | 0.001 | 0.001 | 0.114 |  |  |  | 0.001 | 0.001 | 0.138 |
|  | HDL-C | -0.001 | 0.0004 | 0.118 |  |  |  | -0.001 | 0.0004 | **0.036** |
|  | Triglycerides | -0.003 | 0.001 | 0.021 |  |  |  | 0.003 | 0.001 | **0.017** |
| Resistin | WC | 0.0002 | 0.001 | 0.871 |  |  |  | 0.0004 | 0.002 | 0.785 |
|  | SBP | -0.001 | 0.0004 | 0.131 |  |  |  | -0.001 | 0.0004 | **0.047** |
|  | DBP | 0 | 0.0004 | 0.934 |  |  |  | -0.0002 | 0.0004 | 0.657 |
|  | HbA1c | 0.001 | 0.001 | 0.149 |  |  |  | 0.001 | 0.001 | 0.157 |
|  | HDL-C | 0.001 | 0.001 | 0.068 |  |  |  | 0.001 | 0.001 | 0.139 |
|  | Triglycerides | 0.001 | 0.0004 | 0.078 |  |  |  | 0.001 | 0.0004 | 0.1 |
| L/A | WC | -0.004 | 0.001 | 0.011 |  |  |  | -0.003 | 0.001 | **0.016** |
|  | SBP | -0.001 | 0.002 | 0.432 |  |  |  | -0.001 | 0.002 | 0.4 |
|  | DBP | -0.006 | 0.002 | 0.01 |  |  |  | -0.006 | 0.002 | **0.02** |
|  | HbA1c | -0.003 | 0.002 | 0.223 |  |  |  | -0.002 | 0.003 | 0.433 |
|  | HDL-C | 0.001 | 0.003 | 0.743 |  |  |  | 0.001 | 0.003 | 0.69 |
|  | Triglycerides | 0.003 | 0.004 | 0.526 |  |  |  | 0.002 | 0.004 | 0.595 |
| Model 1: Unadjusted. Model 2: Adjusted for rape exposure. Model 3: Adjusted for baseline MetS and other covariates (age, HIV, alcohol use, smoking, lifetime trauma, depression, and PTSD). | | | | | | | | | | |

Values in bold indicate p-value ≤ 0.05 (unadjusted for multiple testing). * Significant p-value at α*=0.008 (Bonferroni adjustment for multiple testing (6 Mets outcomes for each adipokine marker)) α*= α/no of hypotheses, where α=0.05. Abbreviations: L/A: leptin/adiponectin ratio; WC, waist circumference; SBP, systolic blood pressure; DBP, diastolic blood pressure; HbA1c, Glycated haemoglobin; HDL, high density lipoprotein; MetS, metabolic syndrome

**S9.** **Logistic regression analyses examining the associations of baseline adipokines with Metabolic syndrome (MetS) incidence at 12-month follow-up**

| **Study population** | **Adipokine** | **Nr of Cases/Nr of people at risk**^a^ | **Incidence rates**  **%(95% CI)** | **Unadjusted OR (95% CI)** | **P value** | **Multivariable Adjusted**^b^ **OR (95% CI)** | **P value** |
| --- | --- | --- | --- | --- | --- | --- | --- |
| All participants |  |  |  |  |  |  |  |
|  | Adiponectin | 67/778 | 8.6(6.8 – 10.8) | 0.99(0.94, 1.04) | 0.802 | 1.03(0.97, 1.09) | 0.414 |
| Rape Exposed |  |  |  |  |  |  |  |
|  | Leptin | 23/362 | 6.4(4.3 -9.4) | 1.02(0.99, 1.06) | 0.204 | 0.98(0.93, 1.02) | 0.320 |
|  | Resistin | 23/362 |  | 1.00(0.96, 1.05) | 0.971 | 0.99(0.94, 1.04) | 0.698 |
|  | L/A Ratio | 23/362 |  | 1.06(0.84, 1.33) | 0.646 | 0.58(0.37, 0.92) | **0.021** |
|  | Adiponectin | 23/362 |  | 0.99(0.92, 1.06) | 0.760 | 1.07(0.97, 1.17) | 0.168 |

^a^Excludes participants with MetS at baseline; ^b^Multivariable models are adjusted for age, baseline body mass index, waist circumference, systolic and diastolic blood pressure, high-density lipoprotein cholesterol, HbA1c, triglycerides and HIV-status. Values in bold indicate p-value ≤ 0.05
